# Seasonal variation of mortality from external causes in Hungary between 1995 and 2014

**DOI:** 10.1371/journal.pone.0217979

**Published:** 2019-06-06

**Authors:** Tamás Lantos, Tibor András Nyári, Richard J. Q. McNally

**Affiliations:** 1 Department of Medical Physics and Informatics, University of Szeged, Szeged, Hungary; 2 Institute of Health & Society, Newcastle University, Royal Victoria Infirmary, Newcastle, England, United Kingdom; Universidade Federal de Minas Gerais, BRAZIL

## Abstract

**Objective:**

To analyze trends in external mortality in Hungary between 1995 and 2014 by sex.

**Methods:**

Data on the numbers of deaths due to external causes were obtained from the published nationwide population register. Negative binomial regression was applied to investigate the yearly trends in external-cause mortality rates. Cyclic trends were investigated using the Walter-Elwood method.

**Results:**

Suicide and accidents accounted for approximately 84% of the all-external-cause of deaths in Hungary. Annual suicide, unintentional falls and traffic accidents mortality declined significantly (p-value for annual trend: *p* < 0.001) from 30.5 (95% CI: 29.5–31.5) to 15.8 (15.1–16.5), from 31.2 (30.2–32.2) to 12.2 (11.7–12.8) and from 17.2 (16.4–18) to 5.4 (5–5.8) per 100 000 persons per year, respectively, during the study period. A significant declining trend in annual mortality was also found for assault, cold/heating-related accidents and accidents caused by electric current. However, the declining trend for drowning-related accidents was significant only for males. Significant winter-peak seasonality was found in the mortality rates from accidental falls, cold/heat-related accidents, other accidents caused by submersion/obstruction and other causes. Seasonal trends with a peak from June to July were observed in death rates from suicide/self-harm, accidental drowning/submersion and accidents caused by electric current. A significant seasonal variation with a peak in September was revealed in the mortality due to traffic accidents.

**Conclusions:**

This Hungarian study suggests that there was a significant seasonal effect on almost all kinds of deaths from external causes between 1995 and 2014. Environmental effects are involved in the aetiology of suicide and accidents.

## Introduction

During the period of 20 years between 1995 and 2014, Hungary has recorded (on average) the 4th highest standardised death rates from external causes among the 28 members of the European Union (behind the Baltic states) and the highest one of the Visegrad Group (V4; consisting of the Czech Republic, Hungary, Poland and Slovakia) countries [1]. External causes were one of the most prevalent causes of death in Hungary (annually approximately 7700 deaths on average), ranking fourth with 6.3% of the total mortality among the major causes of death behind circulatory diseases (50.6%), neoplasms (24.9%) and digestive diseases (6.6%)–and ahead of respiratory diseases (4.5%) during this period.

The most common external causes of death in these years have been suicide (nearly 2700 deaths/year on average), followed by unintentional falls (2550) and traffic accidents (1200) which are also the leading external causes of death in many European countries. Additionally, in the last decades, Hungary has registered one of the highest mean mortality rates both from accidental falls and suicide in Europe [1].

Suicide, as one of the external causes of death, has been investigated numerous times and in many ways. Rihmer and colleagues have investigated suicide from clinical-epidemiological perspectives [2]. They reported that completed suicides were decreasing from 1986 to 2006 but this was followed by a stagnant phase. They assumed that the disappearance of the declining trend might be attributable to increasing unemployment and to the cut backs that Hungarian psychiatry faced during those years [3].

Bálint and colleagues have studied socio-epidemiological aspects and found that higher education levels are accompanied by a lower risk of suicide [4]. Marriage was a protective factor against suicide [2].

Rihmer and colleagues reported higher suicide rates in south-eastern Hungary compared with the suicide rates in the north-western parts of the country. Furthermore, a greater level of urbanicity is associated with a lower level of suicide rates. Males in all age groups are more at risk than females and the risk of suicide increases with age [2].

Fountalakis and colleagues investigated 29 European countries between 2000 and 2012 [5]. They found that both economic and climatic factors play a role in the development of suicides, but the impact of the latter is stronger. A significantly higher suicide rate was reported in the late spring–early summer peak in Hungary [6].

Although seasonal variation in mortality from suicide was studied in many countries in Europe and, specifically in Hungary [6, 7, 8], the general cyclic pattern and annual trends of mortality rates from external causes has not been investigated yet. These trends are important as they could lead to preventive measures and greater understanding of the underlying causes.

We hypothesize that cyclic trends could be also detected in other categories of deaths from external causes and that annual trends in mortality rates of external causes of deaths are decreasing.

Previous studies showed that the pattern of mortality from external causes is different among males and females [9] and by age-groups [10]. Therefore, we applied a divided age-group structure and calculated mortality trends by gender.

In our study, the annual and seasonal trends in mortality rates from external causes in Hungary were investigated during the 20-year interval between 1 January 1995 and 31 December 2014.

## Materials and methods

### Study population

Data on the population and numbers of deaths from external causes were obtained from the published nationwide population register of the *Hungarian Central Statistical Office* (HCSO) [11].

The HCSO provided data on the number of births for each month over the study period but by gender only for each year. The number of births in each month for each gender was estimated assuming no monthly variation in the gender ratio within any year.

The data concerning the external cause of deaths have been published online (*Dissemination Database*) by the HCSO [12]. These data were classified according to the International Classification of Diseases, 10th Revision (ICD-10) codes. **S1 Table** summarises the main categories.

Due to similar characteristics, some of the causes of death were grouped together: *traffic accidents* (in the tables, abbreviated as *traffic*) include railway accidents (1), motor vehicle accidents (2) and other transport accidents (3); *cold/heating-related accidents* (abbreviated as *cold-heat*) include accidents caused by smoke, fire and flames (10) and exposure to excessive cold (11); *other causes* (abbreviated as *other*) include other accidents (14) and other external causes of morbidity and mortality (17). Since the numbers of deaths from water transport accidents (4), air transport accidents (5) and lightning (12) were too small (<50), these death causes were added to the category *other causes*.

Data on both the population and numbers of deaths from external causes were classified by age groups as follows: 0–19 years (‘youth’), 20–34 years (‘young adult’), 35–59 years (‘middle-aged adult’) and over 60 years (‘older adults’).

Thus, age-specific death rates were calculated. Furthermore, the mortality rates of external causes were directly standardised [13] using the *European Standard Population* (ESP) published in 1976 [14] to make comparisons easier. The population distribution of ESP is the following: 0–19 years 29%, 20–34 years 21%, 35–59 years 34% and over 60 years 16% of the population.

### Statistical methods

Age-standardised mortality rates (ASMRs) in Hungary during the study period were calculated to make the external-cause mortality rates more comparable over time.

There are several methods for analysing cyclic trends [15]. We have used geometrical models for analysing cyclic variation which was introduced by Edwards [16]. However, Edwards’ method used only the number of observations. Walter and Elwood [17] generalised Edwards’ idea by including the population at risk. They described that seasonal fluctuation of an event which occurs on a fixed date every year might be described using cyclic patterns over the time period. The Walter-Elwood seasonality test has greater power for detecting seasonal trends, thus this test is robust for detecting seasonal effects.

Since the monthly population was estimated, Edwards’ method was applied to confirm the findings of the Walter-Elwood method. Stolwijk and colleagues [18] described the application of generalised linear models (GLMs) for investigating seasonality which is an extension of the Walter-Elwood method and based on similar geometrical approaches. We have applied negative binomial regression [19] to investigate both seasonal and annual trends.

The distribution of rare events is usually skewed and can be well approximated by the Poisson distribution. An important property of this distribution is that the mean is equal to the variance. In this case, the so-called ‘dispersion parameter’ (logarithm of its parameter lambda) is 1. However, in the case of population-level data (e.g. for cause-specific mortality), ‘overdispersion’ is a common phenomenon: the variance exceeds the mean; therefore, the assumptions of Poisson regression are not met. In such cases, negative binomial (NB) regression can be applied.

Since the likelihood-ratio test concerning overdispersion was significant, annual trends were investigated using NB regression models in analyses by gender and type of death from external causes (year of death was the only independent factor included in the model). Incidence rate ratios (IRRs) and 95% confidence intervals (CIs) were calculated.

Additionally, we have used both Walter-Elwood and Edwards’ methods for analysing cyclic variation to confirm the findings. Note that Edwards’ test is sensitive to occasional extreme values; however, both negative binomial regression and Walter-Elwood test are powerful methods in case of extreme values.

Consequently, data on the month of the death were aggregated over the study period and cyclic trends in these monthly data were investigated using the methods mentioned above. Both NB regression and Walter-Elwood test adjust for the population at risk by grouping the data into months and were used to investigate single or double peaks of seasonality.

Analyses were conducted for all causes and for men and women separately. *p*-values less than 0.05 were considered statistically significant. All analyses were performed using STATA Software Version 9.0 (Stata Corp LP, College Station, TX, USA).

## Results

Overall, 154 211 deaths from external causes (66.3% males and 33.7% females) were registered in Hungary during the period 1995–2014. Suicide/self-harm, accidental falls and traffic accidents were the most common causes with 53 769 (34.9%), 51 015 (33.1%) and 24 367 (15.8%) deaths, respectively (**Table 1**).

**Table 1:** Total numbers of deaths by age during the 20 years of study in Hungary.

	Traffic	Falls	Drowning	Other drowning	Electric current	Cold-heat	Alcohol	Other	Suicide	Assault
**OVERALL**										
**0–19**	1362	0	306	62	0	6	0	41	606	48
**20–34**	5525	130	281	25	30	28	0	421	6135	117
**35–59**	10329	7076	1386	2176	188	3239	120	3197	27635	1668
**60-**	7151	43809	540	2685	13	3699	0	3976	19393	808
**Total**	24367	51015	2513	4948	231	6972	120	7635	53769	2641
**Male**										
**0–19**	1034	0	275	53	0	6	0	28	559	28
**20–34**	4947	130	281	25	30	25	0	409	5666	105
**35–59**	8475	6140	1310	1825	185	2772	114	2775	22232	1175
**60-**	4839	17310	450	1503	9	2123	0	2017	13074	382
**Total**	19295	23580	2316	3406	224	4926	114	5229	41531	1690
**Female**										
**0–19**	328	0	31	9	0	0	0	13	47	20
**20–34**	578	0	0	0	0	3	0	12	469	12
**35–59**	1854	936	76	351	3	467	6	422	5403	493
**60-**	2312	26499	90	1182	4	1576	0	1959	6319	426
**Total**	5072	27435	197	1542	7	2046	6	2406	12238	951

As was the case with the crude numbers and age-specific mortality rates, the highest age-standardised rate was also detected in the case of *suicide and self-harm* with an ASMR of 24.06 per 100 000 persons per year (95% CI: 23.86–24.26). The largest number of suicides (more than half of all cases) was found in the age group 35–59 years; however, the highest age-specific mortality rate with 45.07 per 100 000 persons per year was observed in the group aged over 60 years (**Table 2**).

**Table 2:** Age-standardised mortality rates per 100 000 persons in Hungary between 1995 and 2014.

	Traffic	Falls	Drown.	Other drown.	Electric current	Cold-heat	Alcohol	Other	Suicide	Assault
**OVERALL**										
**0–19**	0.879	0.000	0.197	0.040	0.000	0.004	0.000	0.026	0.391	0.031
**20–34**	2.647	0.062	0.135	0.012	0.014	0.013	0.000	0.202	2.940	0.056
**35–59**	5.053	3.461	0.678	1.064	0.092	1.584	0.059	1.564	13.518	0.816
**60-**	2.659	16.292	0.201	0.999	0.005	1.376	0.000	1.479	7.212	0.300
**ASMR**	11.238	19.816	1.211	2.115	0.111	2.977	0.059	3.271	24.061	1.203
**LB (95%CI)**	11.097	19.644	1.164	2.056	0.097	2.907	0.048	3.197	23.857	1.158
**UB (95%CI)**	11.379	19.987	1.258	2.174	0.126	3.047	0.069	3.344	24.264	1.249
**Male**										
**0–19**	1.303	0.000	0.347	0.067	0.000	0.008	0.000	0.035	0.704	0.035
**20–34**	4.468	0.122	0.264	0.023	0.028	0.023	0.000	0.384	5.234	0.099
**35–59**	8.535	6.184	1.319	1.838	0.186	2.792	0.115	2.795	22.390	1.183
**60-**	4.640	16.598	0.431	1.441	0.009	2.036	0.000	1.934	12.536	0.366
**ASMR**	19.127	22.904	2.361	3.369	0.223	4.858	0.115	5.148	40.955	1.684
**LB (95%CI)**	18.857	22.612	2.265	3.256	0.194	4.723	0.094	5.009	40.561	1.603
**UB (95%CI)**	19.397	23.196	2.458	3.483	0.252	4.994	0.136	5.288	41.349	1.764
**Female**										
**0–19**	0.434	0.000	0.041	0.012	0.000	0.000	0.000	0.017	0.062	0.026
**20–34**	0.565	0.000	0.000	0.000	0.000	0.003	0.000	0.012	0.459	0.012
**35–59**	1.763	0.890	0.072	0.334	0.003	0.444	0.006	0.401	5.139	0.469
**60-**	1.405	16.098	0.055	0.718	0.002	0.957	0.000	1.190	3.839	0.259
**ASMR**	4.167	16.988	0.168	1.064	0.005	1.405	0.006	1.620	9.498	0.766
**LB (95%CI)**	4.052	16.787	0.145	1.011	0.001	1.344	0.001	1.556	9.330	0.717
**UB (95%CI)**	4.282	17.189	0.191	1.117	0.009	1.465	0.010	1.685	9.667	0.815

Both in view of crude numbers and age-standardised mortality rates, the second most frequent death cause was *accidental falls* with an ASMR of 19.82 per 100 000 persons per year (95% CI: 19.64–19.99). In the age group below 20 years, there was no death from this cause at all.

This was the only death cause to have had more (even in crude numbers) female victims than male ones (53.8% and 46.2%, respectively). There were many more men among the victims of this external cause in the group aged 35–59 years (the age-specific mortality rates were 18.19 and 2.62 per 100 000 persons per year, respectively). In the age group 20–34 years, there were no female victims of this death cause at all.

However, the excess of deaths from this cause in the above-60 age group was enough for women to ‘surpass’ men even proportionately–even if this difference was not significant (25.12 versus 24.61 per 100 000 persons per year).

The third most prevailing death cause was *traffic accidents* with an ASMR of 11.24 per 100 000 persons per year (95% CI: 11.10–11.38). Although most cases of death due to traffic accidents were registered in the age group 35–59 years, the highest age-specific mortality rate was found in the group aged over 60 years (**Table 2**).

Nearly four-fifths (79.2%) of the traffic accident victims were men and there were (both in crude numbers and proportionately) more male victims in each of the age groups. The difference between males and females was most noticeable in the group aged 20–34 years (22.14 versus 2.69 per 100 000 persons per year).

The next most prevalent external cause (cold/heat-related accidents) was already nearly one order of magnitude smaller (in terms of mortality rates) than the leading one (suicide/self-harm). All the results concerning this death cause, together with the other ones not mentioned above were presented only in tabular form.

### Annual trends in mortality

**Fig 1:**
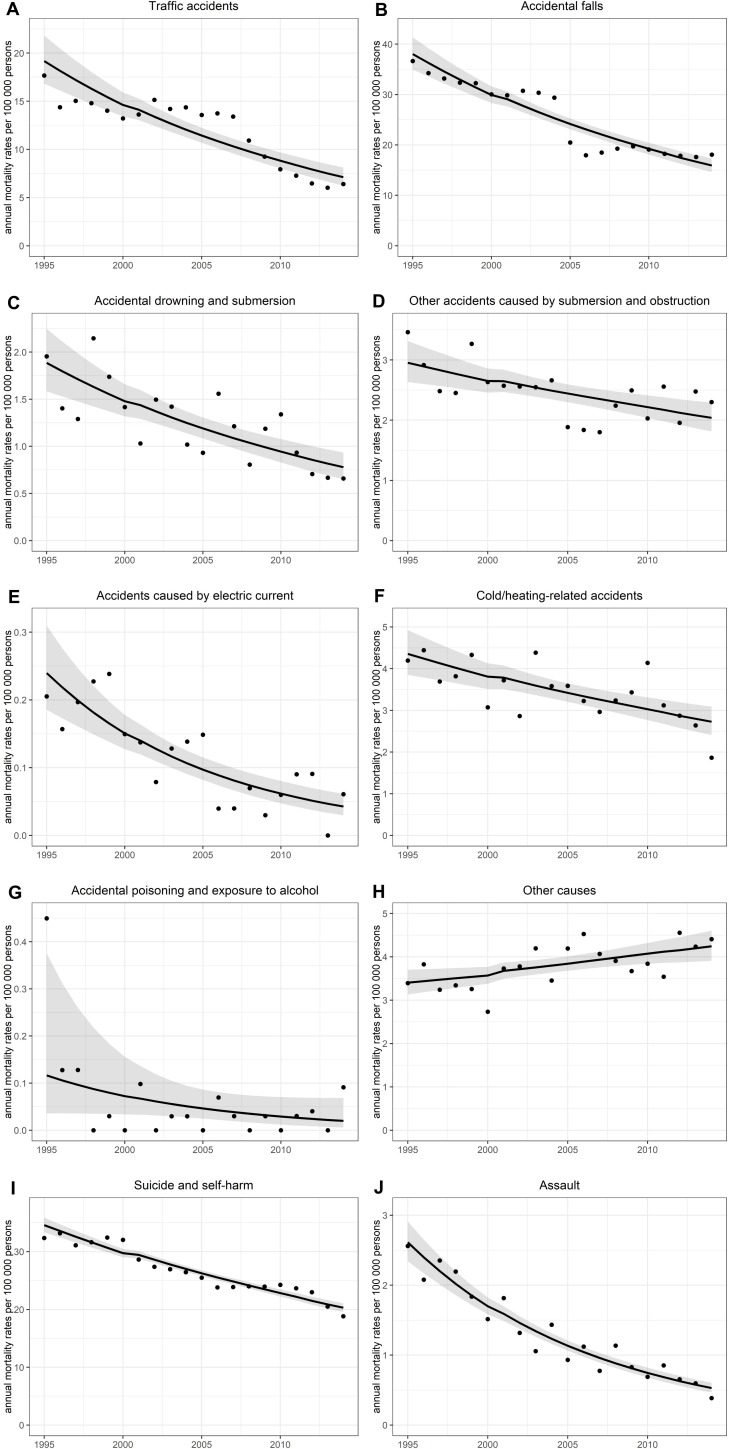
**A-J. Annual trends of deaths from external causes in Hungary between 1995 and 2014.** Annual mortality rates per 100 000 persons: observed (points) and fitted rates (dashed lines) with confidence intervals (grey bands) obtained from negative binomial regression.

The NB regression model for annual age-standardised data revealed a declining trend in the yearly ASMRs for all types of death from external causes except for *other causes* (RR: 1.014, 95% CI: 1.006–1.021; *p* < 0.001) during the study period (**Fig 1A–1H**). However, in the case of *drowning-related death causes* (7–8), the annual trend was significant (*p* < 0.001) only for males. Furthermore, the annual trend for *accidental poisoning and exposure to alcohol* (13) was also significant (*p* = 0.038); however, in the case of males–they accounted for the 95% of the victims–it was no longer significant (*p* = 0.057).

Referring to the leading external death causes, the annual trend was significant (*p* < 0.001) for each one of them. The annual suicide rate declined by 48.2% from the maximum of 30.5 per 100 000 persons in 1995 to the minimum of 15.8 per 100 000 persons in 2014. There was an RR trend per annum of 0.974 (95% CI: 0.971–0.977). A similar decreasing trend was detected for the annual rate of deaths due to accidental falls (from 31.2 to 12.2, by 60.9%) and traffic accidents (from 17.2 to 5.4, by 68.6%) with an annual RR of 0.957 (95% CI: 0.950–0.964) and 0.951 (95% CI: 0.939–0.963), respectively.

Annual trends were displayed by gender (in cases where the results were significant for both sexes) in **S1 Fig**.

### Seasonal trends

The aggregated numbers of deaths are summarised in **Table 3**. The overall number of the deaths from *suicide and self-harm* was the highest among all deaths from external causes. Regarding the overall number of external-cause deaths December was the most frequent month of the year.

**Table 3:** The monthly numbers of deaths used in seasonal analyses in Hungary during the period 1995–2014.

	Traffic	Falls	Drowning	Other drowning	Electric current	Cold-heat	Alcohol	Other	Suicide	Assault	Overall
Jan.	1671	4691	75	448	0	1573	10	726	3838	226	13258
Feb.	1392	3999	97	404	3	1141	16	619	3647	188	11506
Mar.	1611	4340	160	484	0	727	8	633	4758	270	12991
Apr.	1736	4120	129	453	4	312	18	561	4875	223	12431
May	1870	4166	170	382	16	133	8	629	5139	211	12724
June	2077	4206	443	354	81	60	3	607	5010	215	13056
July	2230	4163	639	330	73	59	9	614	5182	215	13514
Aug.	2355	3975	462	328	42	86	6	553	4958	224	12989
Sept.	2328	3956	118	375	9	140	6	631	4581	192	12336
Oct.	2639	4612	54	463	0	382	3	698	4261	209	13321
Nov.	2234	4157	89	407	3	805	20	675	3845	230	12456
Dec.	2224	4630	77	520	0	1554	13	689	3675	238	13620
Total	24367	51015	2513	4948	231	6972	120	7635	53769	2641	154211

Using the Walter-Elwood method, a significant cyclic trend was found in the monthly deaths from each kind of external cause except for *accidental poisoning/exposure to alcohol* (**Table 4**).

**Table 4:** Annual and seasonal trends in Hungary during the period 1995–2014.

	Traffic	Falls	Drowning	Other drowning	Electric current	Cold-heat	Alcohol	Other	Suicide	Assault
OVERALL										
IRR	0.951	0.957	0.956	0.983	0.916	0.978	0.913	1.014	0.974	0.921
95% CI	0.939	0.950	0.941	0.973	0.894	0.966	0.838	1.006	0.971	0.911
	0.963	0.964	0.972	0.993	0.938	0.989	0.995	1.021	0.977	0.931
p for annual trend	<0.001	<0.001	<0.001	0.001	<0.001	<0.001	0.038	<0.001	<0.001	<0.001
peak season	Sep	Dec	June/July	Jan	July	Jan	Jan/Feb	Dec	June	Feb
p for seasonality							NS			NS
Male										
IRR	0.952	0.965	0.955	0.974	0.921	0.975	0.914	1.007	0.977	0.918
95% CI	0.939	0.959	0.941	0.964	0.899	0.964	0.833	1.000	0.973	0.910
	0.964	0.971	0.969	0.984	0.943	0.985	1.003	1.014	0.980	0.926
p for annual trend	<0.001	<0.001	<0.001	<0.001	<0.001	<0.001	0.057	0.039	<0.001	<0.001
peak season	Sept	Oct/Nov	June/July	Jan	July	Jan	Jan/Feb	Nov	June	Apr/May
p for seasonality		NS					NS			NS
Female										
IRR	0.951	0.950	0.979	1.002		0.985		1.029	0.966	0.928
95% CI	0.941	0.948	0.901	0.986		0.972		1.014	0.962	0.918
	0.961	0.952	1.064	1.017		0.999		1.043	0.970	0.939
p for annual trend	<0.001	<0.001	0.621	0.837		0.032		<0.001	<0.001	<0.001
peak season	Sept/Oct	Jan	July	Feb		Jan		Dec/Jan	June/July	Jan
p for seasonality										NS

**Fig 2:**
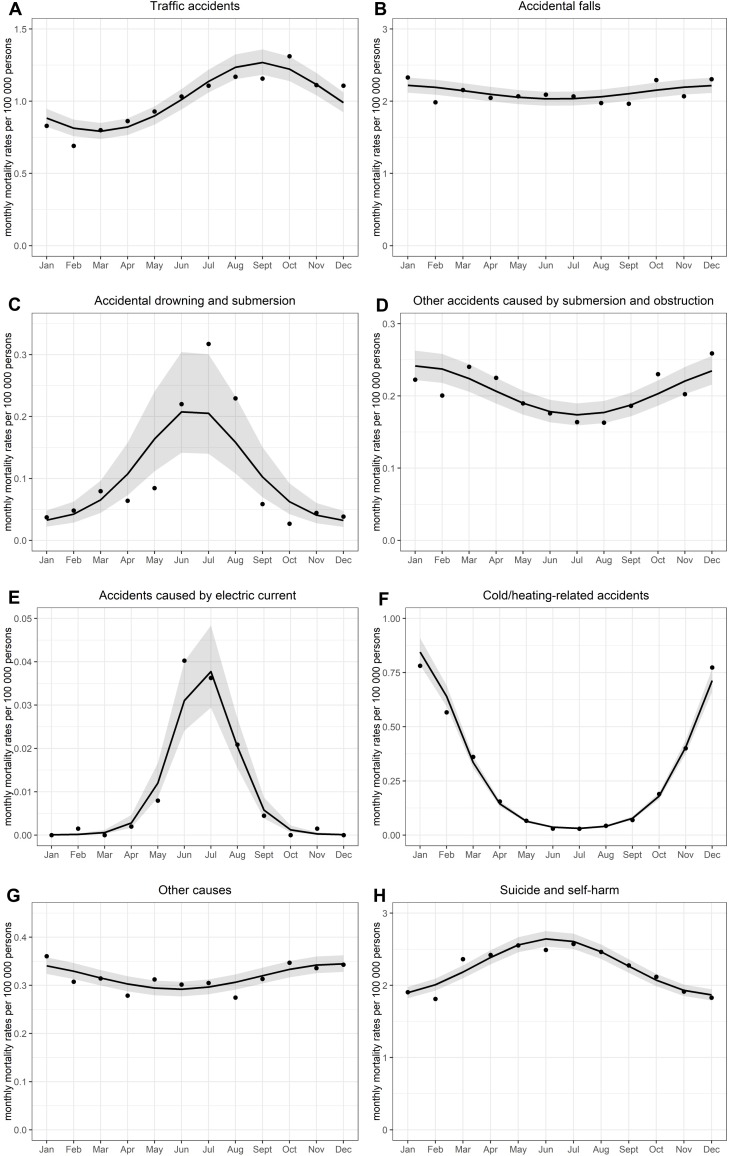
**A–H. Seasonal variation in month of deaths from external causes in Hungary during the period 1995–2014.** Monthly mortality rates per 100 000 persons: observed (points) and fitted rates (dashed lines) with confidence intervals (grey bands) obtained from negative binomial regression.

There was significant seasonality in the mortality rates from *accidental falls* (**Fig 2B**) with a winter peak (peak in December). A winter peak was also detected in the mortality rates from *other accidents caused by submersion/obstruction* (January) (**Fig 2D**) and cold/heat-related accidents (January/February) (**Fig 2F**). There was also a significant peak for *other causes* (in December) (**Fig 2G**); however, it is a heterogeneous group of external death causes and so needs to be treated with caution.

The Water-Elwood method revealed significant seasonality in the mortality rates from *suicide/self-harm* (**Fig 2H**) with a summer peak (June). A summer peak was also observed in the mortality rates from *accidental drowning/submersion* (June/July) (**Fig 2C**) and *accidents caused by electric current* (July) (**Fig 2E**). The mortality rates from traffic accidents peaked significantly in September (**Fig 2A**). However, no more cyclical variation was discovered in the mortality rates either from any other kind of external-cause deaths or from all external causes combined.

It is worth noting that, in the case of accidental falls, the seasonality was significant only for females; for the other types of external death causes, there was no such difference between men and women(**Table 4**).

Seasonal trends were displayed by gender (in cases where the results were significant for both sexes) in **S2 Fig**.

NB regression models confirmed the findings obtained by the Walter-Elwood method. There was no significant double peak model of seasonality.

## Discussion

### Main findings

The leading external causes of death were suicide, unintentional falls and traffic accidents, accounting for more than five-sixths (83.8%) of the mortality from all external causes combined. The number of deaths due to external causes was higher (both in crude numbers and proportionately) for males in all four age groups; the smallest difference (in crude numbers) was found in the above-60 age group, which can be explained by the characteristics of the age pyramid (more females than males in this age group). Overall, this age group accounted for more than half (53.2%) of the external-cause mortality, which was attributable to the large number of fall-related (female) victims aged over 60. However, for males only, the leading age group was the one between 35 and 59 years (due to the large number of deaths from suicide and traffic accidents). Nonetheless, the highest age-specific rate for males was also observed in the group aged over 60.

Either significant seasonal variation peaking in winter or significant summer-peak seasonality was observed in most of the external-cause mortality rates. However, a significant cyclical variation with a peak in September was discovered in the death rates from traffic accidents. Nevertheless, no seasonal trend was found in the combined mortality from external causes.

Decreasing annual trends in the ASMRs for almost all kinds of death from external causes were found during the study period. Consequently, an improving tendency can be observed in Hungary relating to the mortality due to external causes. The annual rate declined by 55.9% from the maximum of 94.4 per 100 000 persons in 1995 to the minimum of 41.6 per 100 000 persons in 2014. This decrease is particularly important since the mortality from external causes is traditionally high in the Central and Eastern European Countries (CEECs).

However, Hungary is still far from the EU-15 average in respect to mortality rates due to external causes (nearly twice higher than the ones in EU-15). Furthermore, the decrease in deaths due to external causes is primarily attributable to the spectacular fall observed in accidental mortality. This is linked to the EU accession in 2004 and the directive that was introduced at this time, which makes it compulsory to install safety belt systems in all types of vehicles that are placed on the market. Nevertheless, we cannot fail to mention that Hungary had switched from manual coding of death causes to machine coding in the same year.

### Strengths and limitations

The data were obtained from vital registers, which could have been influenced by a certain simplification of categorisation during the 20-year interval of the study. Additionally, the official Hungarian population estimates did not account for international migration in the period 1995–2001 (**S1 Appendix**), which caused a sudden increase in population between 1999 and 2000. Although the vital statistics performance index of Hungary is one of the best in the world [20] and the percentage of garbage coded deaths is also quite low in Hungary [21], there can be cause-of-death biases (e. g. youth suicides registered as fatal traffic accidents or fatal falls/accidents due to circulatory diseases). However, we are confident that our results do reflect real trends.

Over-dispersion did not influence our results as the NB regression method was employed in the investigation of annual mortality trend. One of the additional advantages of the present study is the analysis by gender.

The description of incidence rates in terms of seasonal variation or cyclic trends is important in many epidemiological studies since the population-level investigation can be utilised in the prevention. As far as we are aware, this is the first epidemiological study reporting the effect of seasonality for deaths from external causes in Hungary.

Environmental effects are involved in the aetiology of mortality. The available Hungarian monthly mean temperature [22] and precipitation [23] data generally supported these hypotheses for external-cause mortality. Unfortunately, there were no monthly (detailed) environmental data available to investigate causality more precisely. Despite daily temperature data being unavailable, heat-related seasonal effects were found in mortality from suicide, accidental drowning/submersion and traffic accidents.

In the seasonality, monthly cyclic trend analyses, we applied the Walter-Elwood method which is a generalisation of Edwards' test, specifically designed to investigate the seasonality of events with a variable population at risk. The Walter-Elwood's test avoids difficulties relating to the former method: Edwards' test ignored any possibility of variation in the population at risk which might be responsible for the cyclic variation of the disease incidence, the test was sensitive to occasional extreme values [24], it suffered from lack of power for small sample sizes [25] and its assumption of equally spaced time intervals might not be fulfilled in practice. Additionally, the Walter-Elwood seasonality test has greater power for detecting seasonal trends following a sinusoidal pattern than the Pearson's χ^2^ test [26].

### Comparison with other studies

Several studies investigated annual trends in suicide [27, 28, 29]. In concordance with the results of Scandinavian studies, there was a significant decrease in the annual trend during the 20 years of investigation. In Hungary, despite the higher rate of decline, age-standardised suicide rate is still about 1.5 times higher than in the EU overall. Nevertheless, although Eastern Europe (Hungary, Lithuania) has become one of the leading geographical regions based on suicide mortality rates, there is now evidence of an increasing trend in Asia (particularly South Korea) [30].

Rihmer and colleagues investigated suicides from epidemiological and clinical aspects in Hungary, giving recommendations for prevention [2, 31]. Their studies highlighted the role of general practitioners (and health care workers, in general) in prevention: since most of the suicides associated with depression can be prevented, then further training in this area would be important.

In 2012, Hungary had started a major restructuring of its healthcare system; in the next two years, there was a decrease in suicide rates (**Fig 1I**). On the other hand, raising the standard of living, reducing unemployment, improving the quality of health and social care and the proper media communication on depression and suicide go beyond health care. Prevention programs focusing specifically on men would also be needed as they are generally more exposed to and susceptible to social and psychological stress [9]. As far as suicide prevention (especially) for the elderly is concerned, the education for the grieving process would be of high importance.

In a study published in 2015 [32], Majdan and his colleagues conducted research that studied the mortality from accidental falls among the elderly (>65 years) in two countries with a similar population pyramid to Hungary (Slovakia, Austria) between 2003 and 2010. A growing trend was found in Slovakia; however, a declining standardised annual trend was detected in Austria, as we did in Hungary. The difference was explained (at least partially) by the better health system.

In Hungary, a sharp drop in the number of fatal falls was observed in a certain year: from 2004 to 2005 (**Fig 1B**). We might assume that some regulations had a role in this decrease: a new decree on the professional rules for managing local public roads was adopted in 2004, which obliges municipalities to develop winter road management plans [Decree 5/2004 (I. 28.) of the Ministry of Economy and Transport (GKM) on the Professional Rules for the Management of Local Roads].

Hungary used to be the leading one among the countries with highest fall-induced mortality rates in the EU; in 2012, its place was taken by Slovakia in this respect. Consequently, the creation of appropriate regulatory background and its enforcement are important in the prevention of accidental falls.

Both death rates from suicide and falls were higher among the elderly; therefore, the development of a social and health care system that supports older people can be an important element of prevention.

Consistent with the results of other studies [33, 34], there was also a declining trend (especially since 2007; **Fig 1A**) in the mortality from traffic accidents in Hungary. This is likely to be the result of a restriction of the traffic law, with substantial changes in May 2008. One of the main elements of this regulation is that drunk driving is (even in international terms) strictly punishable (“zero tolerance”), and a high fine on the spot can be imposed even in the case of low alcohol consumption.

It is important to note that the 2007 amendment to the law had earned impressive media coverage; thereby, citizens were properly informed about the changes, which could also have contributed to a large decrease in accidents. We speculate that further limitations on speed in the Highway Code would have similar (positive) consequences.

The seasonality of suicide attempts (parasuicide) and completed suicide has been investigated in many countries earlier [27, 29, 35, 36, 37, 38, 39]. We focused on the latter, but the attempts can be also authoritative for suicide in this respect (approximately every tenth attempt is “successful”). The results of the studies mentioned above were the same as ours: late spring–early summer seasonality with a peak in May-June. The previous Hungarian studies [6, 8] came to similar conclusions. This result can be explained by the temperature and mood fluctuations (typical for the elderly) and by the phenomenon “relative unhappiness” caused by the increasing intensity of social activity [40, 41]. Christodoulou and his colleagues stated in their overview that “the seasonal variation is evident in Eastern European countries”; however, they also mentioned that the seasonal association was weakening in Hungary [7, 37].

It would be important for general practitioners to know when suicide peaks: although the summer climax is no longer surprising for researchers in this area, it is much less known among GPs. Of course, not only health professionals are responsible for prevention; raising public awareness on suicide is also essential: since it is still a general misconception that winter holidays are the most depressive period of the year, depressed people get less attention in the summer when they would really need it.

Having investigated the seasonal variation in fall-induced fractures [42] and deaths [43] among the elderly (>65 years), previous studies identified cooler seasons and colder states as risk factors, respectively. This is consistent with the winter peak seasonality (peak in December) we obtained, which can also be explained by the slippery sidewalks and roads due to snow and ice [44].

Several studies on seasonality of injuries and deaths from traffic accidents reported late summer and early autumn seasonality [33, 45, 46]. The September peak obtained in our study is consistent with their results, which can be attributable to the drowsiness (dozing at the wheel, driving mistakes) due to the scorching sun and humidity.

## Conclusions

Our ecological study presented the seasonality pattern of some external-cause mortality which might be related to environmental factors. We found seasonal effects related to–among others–suicide, accidental falls and traffic accidents with peaks in June, December and September, respectively. These death causes account for overwhelming majority of the all-external-cause mortality. Environmental effects are involved in the aetiology of these external causes of death in Hungary.

To reduce the overall mortality rate from external causes and to prolong life expectancy, measures must be taken (e.g. cultural education, paying special attention to the elderly in summer, increased police presence on the roads at the opening of the academic year) to reduce those mortality rates associated with seasonal differences; however, further cohort studies should be carried out to investigate this hypothesis using detailed individual data.

## Supporting information

S1 Table External causes of death. (DOCX)

S2 Table Deviance goodness of fit (GoF) for annual and seasonal trends in Hungary 1995–2014. (DOCX)

S1 Fig A-F. Annual trends of deaths from external causes by gender in Hungary between 1995 and 2014. Annual mortality rates per 100 000 persons for males (red) and females (green): observed (dots/triangles) and fitted rates (dashed lines) with confidence intervals (shaded bands) obtained from negative binomial regression.
(TIF)

S2 Fig A-F. Seasonal variation in month of deaths from external causes by gender in Hungary 1995–2014. Monthly mortality rates per 100 000 persons for males (red) and females (green): observed (dots/triangles) and fitted rates (dashed lines) with confidence intervals (shaded bands) obtained from negative binomial regression.
(TIF)

S1 Appendix Methodology section of “Demographic yearbook 2005”. (PDF)
