# Supplementary material for: Seasonal variation of mortality from external causes in Hungary between 1995 and 2014
Source: PLoS One. 2019 Jun 6;14(6):e0217979. doi: 10.1371/journal.pone.0217979 (PMC6553771; doi:10.1371/journal.pone.0217979)
Supplement: S1 Table — (DOCX) [file pone.0217979.s001.docx]

**S1 Table. External causes of death.**

| N^o^ | Full name | ICD-10 codes | Abbreviated name |
| --- | --- | --- | --- |
| 1 | Railway accidents | V05, V15, V80.6, V81, V87.6, V88.6 | Railway (as part of ‘Traffic’) |
| 2 | Motor vehicle accidents | V02-04, V09.0, V09.2, V12-14, V19.0-2, V19.4-6, V20-79, V80.3-5, V86, V87.0-5, V87.7-8, V88.0-5, V88.7 | Motor vehicle (as part of ‘Traffic’) |
| 3 | Other transport accidents | V01, V06, V09.1, V09.3, V09.9, V10-11, V16-18, V19.3, V19.8-9, V80.0-2, V80.7-9, V82-85, V87.9, V88.9, V89.1, V89.3, V89.9, V98-99 | Other transport (as part of ‘Traffic’) |
| 4 | Water transport accidents | V90-94 | Water transport |
| 5 | Air transport accidents | V95-97 | Air transport |
| 6 | Accidental falls | W00-19 | Falls |
| 7 | Accidental drowning and submersion | W65-74 | Drowning |
| 8 | Other accidents caused by submersion and obstruction | W75-84 | Other drowning |
| 9 | Accidents caused by electric current | W85-87 | Electric current |
| 10 | Accidents caused by smoke, fire and flames | X00-09 | Smoke-fire-flame (as part of ‘Cold-heat’) |
| 11 | Exposure to excessive cold | X31 | Cold (as part of ‘Cold-heat’) |
| 12 | Lightning | X33 | Lightning |
| 13 | Accidental poisoning and exposure to alcohol | X45 | Alcohol |
| 14 | Other accidents | W00-X59 difference, Y40-Y84 | Other accidents (as part of ‘Other’) |
| 15 | Suicide and self-harm | X60-84 | Suicide |
| 16 | Assault | X85-Y09 | Assault |
| 17 | Other external causes of morbidity and mortality | Y10-36, Y85-89 | Other external (as part of ‘Other’) |
