# Supplementary material for: Seasonal variation of mortality from external causes in Hungary between 1995 and 2014
Source: PLoS One. 2019 Jun 6;14(6):e0217979. doi: 10.1371/journal.pone.0217979 (PMC6553771; doi:10.1371/journal.pone.0217979)
Supplement: S2 Table — (DOCX) [file pone.0217979.s002.docx]

**S2 Table.** **Deviance Goodness of Fit (GoF) for** **annual and seasonal trends in Hungary 1995-2014.**

|  | Traffic | Falls | Drowning | Other drowning | Electric current | Cold-heat | Alcohol | Other | Suicide | Assault |
| --- | --- | --- | --- | --- | --- | --- | --- | --- | --- | --- |
| OVERALL | | | | | | | | | | |
| p for annual trend | <0.001 | <0.001 | <0.001 | 0.001 | <0.001 | <0.001 | 0.038 | <0.001 | <0.001 | <0.001 |
| Deviance GoF | 0.327 | 0.329 | 0.333 | 0.325 | 0.060 | 0.315 | 0.222 | 0.322 | 0.321 | 0.270 |
| p for seasonality |  |  |  |  |  |  | NS |  |  | NS |
| Deviance GoF | 0.209 | 0.214 | 0.204 | 0.227 | 0.003 | 0.308 |  | 0.203 | 0.210 |  |
| Male | | | | | | | | | | |
| p for annual trend | <0.001 | <0.001 | <0.001 | <0.001 | <0.001 | <0.001 | 0.057 | 0.039 | <0.001 | <0.001 |
| Deviance GoF | 0.326 | 0.318 | 0.353 | 0.323 | 0.073 | 0.304 |  | 0.321 | 0.323 | 0.265 |
| p for seasonality |  | NS |  |  |  |  | NS |  |  | NS |
| Deviance GoF | 0.204 |  | 0.205 | 0.223 | 0.001 | 0.159 |  | 0.260 | 0.209 |  |
| Female | | | | | | | | | | |
| p for annual trend | <0.001 | <0.001 | 0.621 | 0.837 |  | 0.032 |  | <0.001 | <0.001 | <0.001 |
| Deviance GoF | 0.330 | 0.328 |  |  |  | 0.322 |  | 0.314 | 0.336 | 0.229 |
| p for seasonality |  |  |  |  |  |  |  |  |  | NS |
| Deviance GoF | 0.216 | 0.216 | 0.120 | 0.228 |  | 0.636 |  | 0.196 | 0.205 |  |
