# Supplementary figures and images for: Seasonal variation of mortality from external causes in Hungary between 1995 and 2014
Source: PLoS One. 2019 Jun 6;14(6):e0217979. doi: 10.1371/journal.pone.0217979 (PMC6553771; doi:10.1371/journal.pone.0217979)

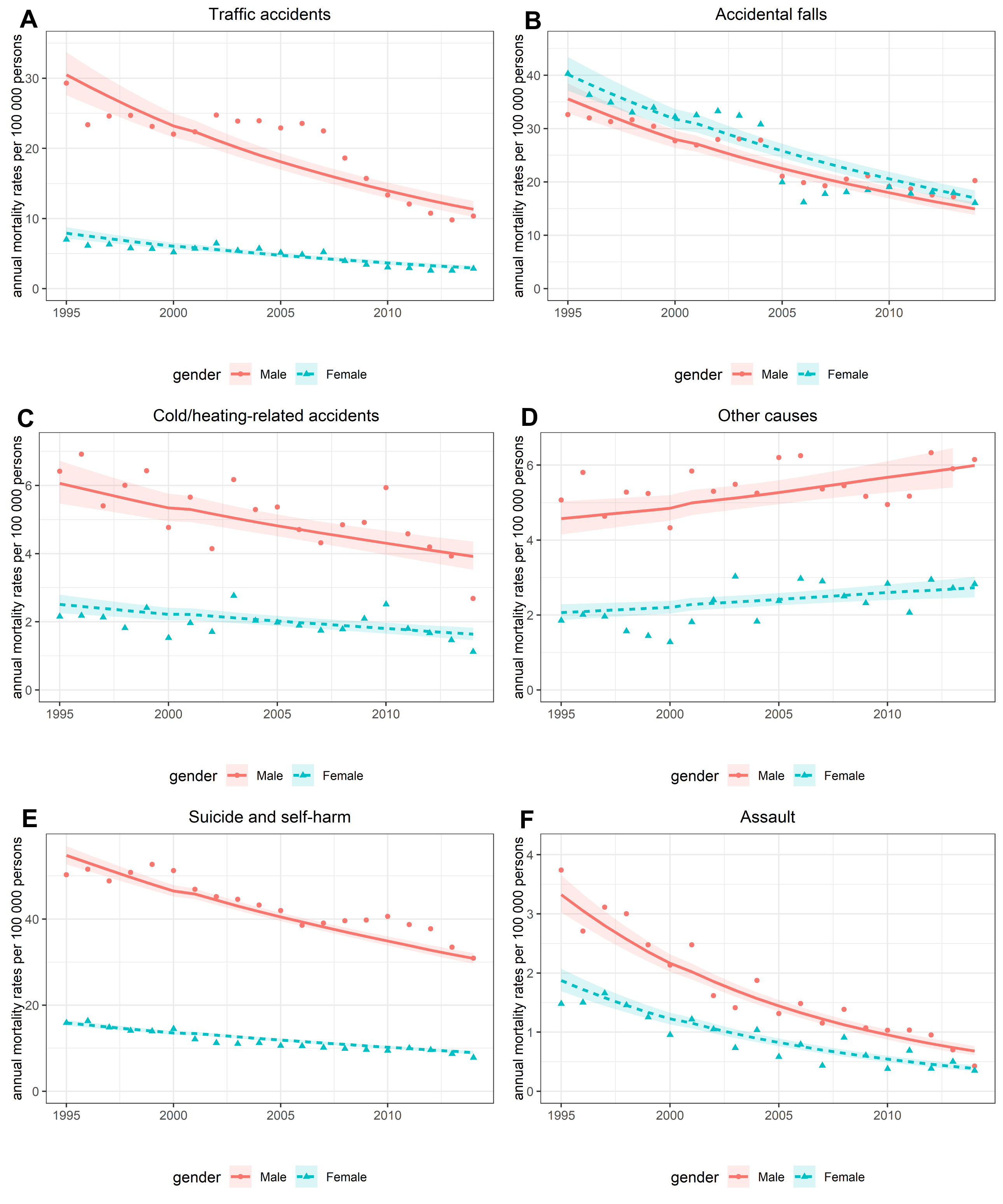

Supplement: S1 Fig — A-F. Annual trends of deaths from external causes by gender in Hungary between 1995 and 2014. Annual mortality rates per 100 000 persons for males (red) and females (green): observed (dots/triangles) and fitted rates (dashed lines) with confidence intervals (shaded bands) obtained from negative binomial regression. (TIF) [file pone.0217979.s003.tif]

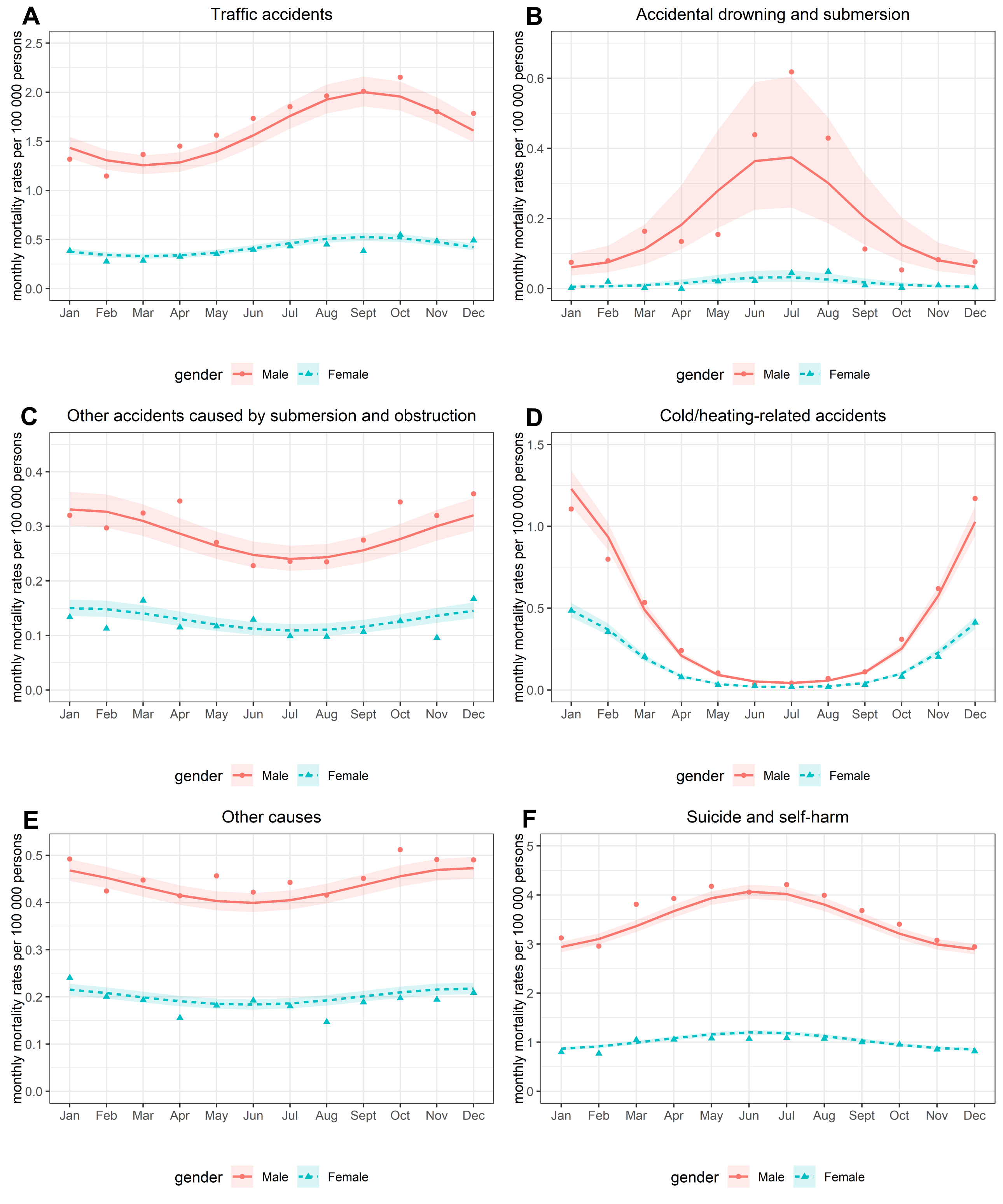

Supplement: S2 Fig — A-F. Seasonal variation in month of deaths from external causes by gender in Hungary 1995–2014. Monthly mortality rates per 100 000 persons for males (red) and females (green): observed (dots/triangles) and fitted rates (dashed lines) with confidence intervals (shaded bands) obtained from negative binomial regression. (TIF) [file pone.0217979.s004.tif]
