# Supplementary material for: Seasonal variation of mortality from external causes in Hungary between 1995 and 2014
Source: PLoS One. 2019 Jun 6;14(6):e0217979. doi: 10.1371/journal.pone.0217979 (PMC6553771; doi:10.1371/journal.pone.0217979)
Supplement: S1 Appendix — (PDF) [file pone.0217979.s005.pdf]

GENERAL INFORMATION

Data on the population and vital events refer to the present area of Hungary.

Population number

Population numbers have been stated on basis of the data of the population censuses.

Till 1900 population censuses took into account the civil population *and after that date* – total population.

Till 1944 the population numbers also contained the data of three villages (Dunacsun, Horvátjárfalu, Oroszvár) of the Pozsony (Bratislava) bridge head. *In the period between the 1910 and 1941 population censuses the total population number of these villages varied between 3000 and 4000.*

Up to 1970 data refer to the population present *(according to the "de facto" principle the persons staying in the respective administrative unit on the census day), after 1970 to the resident population. (Data are indicated in a comparative structure.)*

At the statement of the yearly population of the inter-census period *the starting-point is the final population number of the last population census for the continuous registration of which the data of natural increase and decrease, respectively, available from vital statistics were used till 2000.*

From 1 January 2001 on the method of the continuous registration changed. The essence of the change is that in the future we take into consideration the international migration too at the statement of the population number between two population censuses. The starting data consist of two subgroups: on the one hand of the resident population of the Hungarian citizens in Hungary according to the population census, on the other hand of the resident population of the foreign citizens in Hungary according to the administrative registrations.

First the population number of 1 January 2001 was stated from the final data of the population census carried out on 1 February 2001. The live births of children of Hungarian citizenship registered in Hungay, the deaths, marriages, divorces, internal migration (permanent and temporary) difference of Hungarian citizens, as well as the international migration balance of the Hungarian citizens (the yearly difference in the number of foreigners having got a Hungarian citizenship and in the number of persons who gave up the Hungarian citizenship, the yearly difference in the number of returning Hungarian citizens and the emigrating Hungarian citizens and the yearly difference in the number of Hungarian citizens leaving for a temporary stay and returning from it) represent the number of the Hungarian population at the beginning of the next year. The number of foreigners having a residence permit, an immigration permit or a settlement permit and refugees, who have a registered adress in Hungary are available from the registrations of the Ministry of the Interior and this is the number of foreign population of 1 January.

The continuous registration of the female population *by birth year, marital status and number of children starts from the data of the population census. The general method of the continuous registration has been changed from 1 January, 2001 (see above). The continuous registration of the females of Hungarian citizenship by number of children is carried out on basis of the yearly data of births, deaths, marriages and divorces occurred in Hungary. In case of the females of foreign citizenship staying in Hungary the basis of the continuous registration by number of children is the distribution by number of children of the Hungarian females of respective birth year and marital status. The two subgroups together form the number and the composition of the female population of Hungary by birth year, marital status and number of children on 1 January of the given year.*

The population number of the regional units *(regions, counties, towns, communes) is stated according to the concept of the resident population: the total number of Hungarian citizens having a place of residence on the given area and having no place of stay elsewhere as well as of Hungarian citizens having a place of stay on the same area and of the resident foreign citizens and refugees according to the registrations of the Ministry of the Interior.*

Vital events

Determination of vital statistical data for years 1876–1918 was based on estimates. It took into consideration not only the data of invariably integral counties and municipal towns, but also those of counties belonging to the country only partly, in a share equal to the former proportion of population to the total territory of the county. Between 1876 and 1885 the shares were stated on basis of the population rate valid at the end of 1880, between 1886 and 1895 – at the end of 1890, between 1896 and 1905 – at the end of 1900, and from 1906 on basis of the population rate at the end of 1910. With this method we stated the number of live births and deaths till 1900, that of marriages till 1910, that of divorces till 1915 and the number of infants deceased at the age under 1 year till 1918 inclusively.

Data on marriages, births and deaths from 1911 to 1946 and the numbers of divorces and those of infants deceased below the age of 1 year between 1919 and 1946 also included data of the three villages (Dunacsun, Horvátjárfalu, Oroszvár) of the Pozsony (Bratislava) bridge-head.

Till 1895 the data of vital events refer to the civil, *from 1896 to the* total population.

The national and regional (region, county, town, village) net rates by years of age, age-groups and sex for the years 1990 and 2000 were calculated using mid-year resident population corrected on the basis of the 2001 population census.

Standardization has been used for comparison of vital statistical rates over time. At calculations the direct method of standardization has been employed, where the standardized rate (m_s) was indicated as the ratio of the actual vital rates by age (m_x) weighted with the standard age-structure of population (P^s_x).

$$m_s = \frac{\sum_{x=0}^{\omega} m_x P_x^s}{\sum_{x=0}^{\omega} P_x^s}$$

See more detailed description of standardized vital statistical rates at the respective vital event.

The date of vital events always refer to the date of occurance.

Data –except for some international data – are final.

Classifications, groupings

Groupings concerning economic activity, occupation *follow the groupings of the 2001 population census.*

The educational qualification *was stated on basis of the 2001 population census taking into consideration of the grades (years) completed in the education of school system.*

Grouping of regional data: *ranking by regions, counties, towns of county rank, towns, villages - if not otherwise indicated - corresponds to the administrative division of 1 January 2004. If there is no comment on the regional tables, then the line "towns" does not contain the data of the capital, the lines "towns" and "villages" exclude data of foreigners, homeless and of those whose residence is unknown.*

The unknown data - *if they don't form a separate group and if there is no other comment, respectively - are ranked as follows:*

- *in case of age the oldest age, age-groups;*
- *in case of duration of marriage the group of the longest duration of marriage;*
- *at the grouping of divorces, females deceased by number of children the highest group of the number of children;*
- *in case of divorces at the grouping by number of divorces of males and females the first divorce;*
- *in case of marital status the group of single males and single females;*
- *in case of birth order the highest group;*
- *in case of infants deceased the group over 2500 grams.*

POPULATION

(Tables 1.1.1–1.1.12., 2.1.1–2.1.10., 3.1.1–3.1.10.)

Population present: *number of persons staying at the respective administrative unit at the census moment. The present population of the country does not include the Hungarian citizens living (staying) abroad but it includes (except the members of diplomatic corps) the foreign civil persons staying in the country.*

Till the 1970 population census the enumeration took into consideration the present population. However the distribution of the present population by administrative units - because of the great temporary movement between the units - may cause a significant difference at the calculation of various indicators. Naturally also the population numbers calculated continuously by administrative units between the two population censuses on basis of the present population of the population censuses are similarly imperfect. It seemed more expedient to calculate the indicators for a population which takes into consideration the attractive or pushing effect - in economic respect - of the respective area,

administrative unit. That is why the international recommendations suggest to use the number of the permanent and resident population for the calculation of the regional indicators, according to local demands.

Permanent population: *number of persons having a residence on the respective area.*

Resident population: *the total number of persons having a residence on the respective area and having no place of stay elsewhere as well as of persons having a place of stay on the same area.* Place of residence: *the address of the dwelling in which the citizen lives. From the point of view of the registration of the home address the following can be considered as a dwelling: a building or a part of building, consisting of one or more living premises, used by the citizen as home as well as the room where somebody lives in need or lodges if he/she has no other dwelling (Act LXVI of 1992 on the registration of the personal data and home address of the citizens, §.5. /2/). This corresponds to the earlier permanent place of residence.* Place of stay: *the address of the dwelling where a person stays longer than three months without the intention to leave finally place of residence (Act LXVI of 1992 on the registration of the personal data and home address of the citizens, §.5. /3/). This corresponds to the former temporary place of residence.*

Natural increase (decrease): *the difference between live births and deaths.*

Difference in permanent migration: *the difference between the number of persons registered with a permanent character to an administrative unit and of those who were registered with a permanent character to another administrative unit from there.*

Difference in temporary migration: *the difference between the total number of persons registered with a temporary character to a respective administrative unit as well as of those registered temporarily elsewhere but returning to the respective administrative unit as their permanent residence and the total number of those who are registered temporarily to another administrative from the respective unit as well as of those being in another administrative unit and returning to their permanent place of residence.*

Actual increase (decrease): *the sum of natural increase/decrease and of the migration (internal and international) difference (+,–).*

Mid-year population (P_k): *the arithmetic mean of the population number at the beginning of the year (P_1) and at the end of the year (P_2). Its calculation:* $P_k = \frac{P_1 + P_2}{2}$

Average annual increase, decrease (–): *between two population censuses the average annual increase or decrease (last column of Table 1.1.1.) was calculated with the assumption that between two population censuses the population increases according to a geometric progression, i.e. the ratio of annual increase is constant.*

Average age: *the weighted arithmetic mean age of living population at a point of time.*

Dependency ratio: *the child (0–14 years) and old age population (65–X years) as a percentage of population aged 15–64 years.*

Ageing index: *the old-age population (65–X years) as a percentage of the child population (0–14 years).*

Data by regional units: *the population data of 1 January 2006 by regions (region, county, town, village) and by settlement population were grouped according to the administrative division of 1 January 2006.*

Source of data: *the comprehensive population census carried out by the Hungarian Central Statistical Office every ten years, the statistics of vital events (marriages, divorces, live births, deaths), and in case of the internal migration the data collection based on §.10. of the 1993 Act No XLVI and besides data of international migration on foreigners and Hungarian citizens the various registrations of the Ministry of the Interior.*

MARRIAGE

(Tables 1.1.13., 1.2.1.–1.2.11., 2.2.1.–2.2.21., 3.1.11– 3.1.13., 3.2.1.–3.2.5.)

Marriage: *contracted before the registrar acting officially in the presence of two witnesses.*

Crude marriage rate: *the ratio of contracted marriages in a given year to thousand midyear population.*

Marriage rate (net): *the ratio of married males and females aged 15 years and over to thousand midyear population. Non-married population covers single, widowed and divorced population aged 15 years and over, who are ready for marriage.*

The balance of marriages: *comparison of the number of marriages and of the number of marriages ceased through the death of a married party or by a judge's decision; the difference between the number of marriages and the number of the marriages ceased. In the balance of marriages the joint impact of the marriage movement and deaths is reflected.*

Average age at marriage: *a weighted arithmetic mean calculated on the basis of marriages by age in the given calendar period. Is calculated by adding 0.5 year to the completed age of the marrying people, presuming an even distribution of marriages in the given year. Because of changes in methodology the average calculated with the new method is higher by 0.5 year on average than the data of 1997 and of earlier years published in the 2001 and former yearbooks.*

Average age at first marriage: *a weighted arithmetic mean of persons (single men and women) by age marrying for the first time in the given calendar period.*

Total first marriage rate: *an indicator showing what share of males and females entering the marrying age of 15 years would contract marriage by a certain age (females 49 years, males 59 years). It rests on the supposition that females and males reaching 15 years of age would show the same marrying disposition up to their age of 50 and 60 years, resp. as the rate of the given year.*

Remarrying person: *the one whose marital status was widowed or divorced before the contract of marriage.*

Duration of marriage: *the period from the date of marriage to the date when the judge's decision comes into force or to the start of the eventual widowhood, respectively.*

Standardized crude marriage rate: *a crude marriage rate calculated with the age-structure of indicated population census years. The population by age-groups deriving from censuses was taken as standard weight, while the rates by age-groups are the ratio of the contracted marriages and the population of the age-group in the given year. Relying on the abovesaid the number of standardized marriages is the half of the product of multiplication of rates by age-groups and standard weights. At the calculation of standardized crude marriage rates the following age-groups were formed: 0–14, 15–19, 20–24, 25–29, 30–34, 35–39, 40–49, 50–59, 60–X ages.*

Standardized net marriage rate: *marriages per thousand non-married males (females) aged 15 years or older. The non-married male (female) population of the indicated census age-group was taken as standard weight, while rates by age-groups are the ratio of male (female) marriages in the given year and of the number of non-married male (female) population. On the basis of the above the number of standardized male (female) marriages is the sum of the product of standard weights and rates by age-groups. At the calculation of the standardized net marriage rates the following age-groups were formed: 15–19, 20–24, 25–29, 30–34, 35–39, 40–49, 50–59, 60–X ages.*

Grouping of the regional data: *till 1970 inclusively the basis of the grouping was the last place of residence of the wife, from 1971 on the last actual place of residence of the wife.*

The marriages ceased through death *do not cover the data of persons of unknown marital status.*

Source of data: *the data collection of the Hungarian Central Statistical Office called Marriage Record carried out on basis of §.10. of the 1993 Act No. XLVI.*

DIVORCE

(Tables 1.1.13., 1.3.1.–1.3.9., 2.3.1.–2.3.21., 3.1.11.– 3.1.13., 3.3.1.–3.3.5.)

Divorce: *a marriage dissolved or annulled by a court's decision entered into legal force. A court's decision dissolving or annulling the marriage enters into legal force if no further legal remedy can be raised against it.*

A factor limiting the comparison is *that till 1958 the data of divorces contained the number of marriages dissolved and annulled by a final judgement in the year under consideration, from 1959 on the number of marriages dissolved and annulled by a court's decision having entered into legal force in the given year. Till 1957 the date of accounting of the data of divorces by age and duration of marriage was the date of commencing a legal action, in 1958–1959 the date of the valid decision, from 1960 on the date when the decision entered into legal force.*

Crude divorce rate: *the ratio of divorces in a given year to thousand midyear population.*

Divorce rate (net): *the ratio of divorced males and females aged 15 years and over to thousand midyear population.*

Average age at divorce: *a weighted arithmetic mean calculated from the age-specific divorces of the divorced people in the given calendar period. At the calculation we add 0.5 year to the completed age of age of divorcees, presuming an even distribution of divorces in the given year. Because of changes in methodology the average age calculated with the new method is higher by 0.5 year on average than the data of 1997 and of earlier years published in the 2001 and former yearbooks.*

Average duration of marriage at divorce: *the average period from contracting a marriage to divorce is calculated as an weighted arithmetic mean of divorce rates.*

Total divorce rate: *it shows how many divorces would fall on marriages contracted in the reviewed period if the marriage-duration-specific divorce rates would prevail in the given calendar period (e.g. year).*

Child born from a dissolved marriage: *total number of common children born alive from the divorced parties' marriage dissolved at present and from their cohabitation prior to the marriage, inclusive of the children deceased in the meantime.*

Living children born from the dissolved marriage: *the number of living children deriving from the divorced parties' marriage dissolved at present and from their cohabitation prior to marriage also contains the number of major children having left the family.*

Liveborn children of the divorced male, female: *total number of the divorced parties' children born alive till the date of coming into legal force of the decision, irrespective of the fact whether the children were born from the marriage dissolved at present, from a previous marriage or out of wedlock.*

Number of living children: *number of common living children deriving from the divorced couple, irrespective of the legal duration of marriage (in 1956 of the date of commencing a legal action; in 1960 and 1970 the number of common children born during the legal duration of the marriage dissolved was indicated.)*

Standardized crude divorce rate: *crude divorce rate calculated with the age-structure of given population census years. The population of the indicated census age-group was taken as standard weight, while rates by age-groups are the ratio of dissolved marriages and of population of the respective age-group in the given year. Relying on the abovesaid the number of standardized divorces is the half of the product of multiplication of rates by age-groups and standard weights. At the calculation of standardized crude divorce rates the following age-groups were formed: 0–14, 15–19, 20–24, 25–29, 30–34, 35–39, 40–49, 50–59, 60–X ages.*

Standardized net divorce rate: *divorces per thousand married males (females) aged 15 years or older. The married male (female) population of the indicated census age-group was taken as standard weight, while rates by age-groups are the ratio of male (female) dissolved marriages in the given age-group and of the number of married male (female) population. On the basis of the above the number of standardized male (female) divorces is the sum of the product of standard weights and rates by age-groups and sexes. At the calculation of the standardized net divorce rates the following age-groups were formed: 15–19, 20–24, 25–29, 30–34, 35–39, 40–49, 50–59, 60–X ages.*

At the grouping of the regional data *till 1955 the basis of grouping was the seat of the court delivering the decision of first instance, from 1956 to 1970 the last place of the common permanent residence of the divorcees and from 1971 on the last actual place of residence of the wife.*

Source of data: *the data collection of the Hungarian Central Statistical Office called Divorce Record carried out on basis of §.10. of the 1993 Act No. XLVI.*

LIVE BIRTH

(Tables 1.1.13., 1.4.1.–1.4.15., 2.4.1.–2.4.29., 3.1.11.– 3.1.13., 3.4.1.–3.4.5.)

Live birth: *(according to the recommendation of the UN) a foetus is live-born if it gives any sign of life after birth, regardless of the length of pregnancy and the length of life after birth.*

Crude live birth rate: *the ratio of live births of a given year to thousand midyear population.*

Live birth rate: *live births per thousand females belonging to the same age-group as the mother.*

Standardized crude live birth rate: *crude live birth rate calculated with the age-structure of indicated population census years. The population by age-groups deriving from censuses was taken as standard weight, while the rates by age-groups are the ratio of the number of children born alive to females in the given age-group of the selected year and of the female population of the same age-group. Relying on the above said the number of standardized live births is the sum of the product of live birth rates by age-groups and standard weights. At the calculation of standardized crude live birth rates the following age-groups were formed: 0–14 year-old population, 15–19, 20–24, 25–29, 30–34, 35–39, 40–44, 45–49 year-old female population; 15–49 year-old male population; 50–X year-old population.*

Standardized net live birth rate: *live births per thousand females aged 15–49 years. The female population aged 15–49 years of indicated censuses was taken as standard weight, while the rates by age-groups are the ratio of the number of liveborn children in the given female age-group and of the female population of the age-group in the given year. On the basis of the above the number of standardized live births is the sum of the product of live birth rates by age-groups and standard weights. At the calculation of standardized net live birth rates the female population was grouped by five years between the age of 15–49 years.*

Fertility indicators. Total fertility rate: *it expresses to how many children a female would give birth during her life at the birth frequency by age of the given year.* Crude (gross) reproduction rate: *it shows to how many daughters a female would give birth during her life at a birth frequency by age of the given year.* Net reproduction rate: *it indicates how many daughters per female would reach the childbearing age at the mortality rate of the given year. If the value of the indicator is 1, this shows the stagnation of the population, a value over 1 means an increase and a value under 1 a decrease.*

Live birth order: *the numerical order of birth of the mother's new-borns. In case of multiple birth every child must be taken into account by order of their birth.*

Average age of child-bearing females: *the average age is indicated with a weighted arithmetical mean calculated on basis of the age-specific live birth data of the females giving birth to children in the given calendar period. At the calculation we add 0.5 year to the completed years of age of child-bearing females, supposing an even distribution of child-births in the given year. The average age of the child-bearing females can be calculated by live birth order, too.*

Formula: $$\bar{x}_i = \frac{\sum_x (x + 0{,}5) \cdot B_{xi}}{\sum_x B_{xi}},$$

where

x – age of the females completed at the date of child-birth,

B_{xi} *– the number of live births No. i of the given females of age x.*

At the data indicated in the 1997 and former yearbooks the average age calculated with the new method is higher by 0.5 year.

Supporter: *in case of a marital or cohabiting partnership the male, in case of a mother bringing up her child alone the female, in case of dependants the private person who provides for their living (parent, relative etc.) or an institutional supporter.*

At the grouping of the regional data *till 1970 the basis of grouping was the mother's last permanent place of residence, from 1971 on the mother's last actual place of residence. In case of mothers who have only a place of residence (a permanent registered dwelling) and have no place of stay (temporary registered dwelling) the administrative unit where they have or had, respectively their last place of residence, in case of those who beside their*

place of residence (permanent registered dwelling) have a place of stay too (temporary registered dwelling) the administrative unit where they have or had, respectively, their last place of stay.

Source of data: *the data collection of the Hungarian Central Statistical Office on the Live Birth Record carried out on basis of §.10. of the 1993 Act No. XLVI.*

FOETAL LOSS

(Tables 1.5.1–1.5.7., 2.5.1.–2.5.23., 3.5.1.–3.5.4.)

Foetal loss: *foetal deaths and induced abortions together.*

Foetal death: *death of the foetus before the delivery (complete expulsion or extraction from the mother) irrespective of the duration of pregnancy. The death is indicated by the fact that the foetus does not breathe or show any other sign of life, such as function of the heart, pulsation of the umbilical cord, movement of the voluntary muscles.* Early and medium-term foetal death: *abortion after a pregnancy shorter than 22 complete weeks (early) and a pregnancy of 22–24 complete weeks (medium-term), respectively, i.e. the spontaneous and late abortion, the mola and extra-uterine pregnancy as well as abortion resulting from another anomaly of conception.* Late foetal death: *if after the separation from the mother's body the foetus did not show any sign of life and if from the conception longer than 24 (till 1996 28) complete weeks passed or if the age of the foetus cannot be stated, the length of the foetus is 30 cm or more or if the weight of the foetus is 500 g or more.*

Induced abortion: *interruption of pregnancy through intentional – surgical – intervention.*

The 1992 Act No. LXXIX on the protection of foetal life modified the earlier motives – valid since 1973 – of the approvals for induced abortions. According to this it is allowed to interrupt a pregnancy if it seriously endangers the health of the female or the foetus, if the pregnancy is the consequence of a crime or if the female is in a grave crisis situation.

According to the definition of the Decree of the Ministry of Health No 18/2000(June 29) a grave crisis situation occurs when it causes bodily or mental impairment or socially intolerable situation.

Abortion rate: *induced abortions per thousand females belonging to the same age-groups as the female.* Total abortion rate: *it expresses to how many induced abortions a female would undergo during her life if the abortion frequency by age of the given year.*

Source of data: *data collections concerning the induced abortions and foetal deaths are based on the data supply of hospitals.*

From 1 January 1996 the data supply on abortions is carried out on the questionnaire called "Record on foetal death" on basis of §.10. of the 1993 Act No. XLVI.

Since 1 January 1993 the source of data of induced abortions is the questionnaire No. 1165 OSAP called "Record B for the statistical data supply on the induced abortion" compulsory on basis of the governmental regulation No. 152/1992/XII.20./. This questionnaire was prepared taking into consideration the 1992 law which modified the earlier motives of approvals of the induced abortions (See in detail also the headword Induced abortion.)

The groupings *according to the various criteria are the summarised results of the above mentioned data collections as well as of the data of the questionnaires sent to the Hungarian Central Statistical Office.*

DEATH

(Tables 1.1.13., 1.6.1.–1.6.20., 2.6.1.–2.6.49., 3.1.11.–3.1.13., 3.6.1–3.6.15.)

Death: *final passing away of all signs of life at any time after live birth at any time, i.e. cessation of all life-functions without the capability of revival.*

The rates of deceased infants at the age of 0 year *(tables 1.6.4–1.6.6., 2.6.2.–2.6.4., 2.6.7., 2.6.20–2.6.22., 3.6.5., 3.6.6., 3.6.7.) are calculated for live-born.*

Standardized death rate: *crude death rate calculated with the age-structure of given census years, or with that of the european standard population according to WHO. Population in five-year age-groups of given censuses or of the WHO european standard were taken as standard weights, while rates by age-groups are the ratio of deaths and population number of the relevant age-group. On the basis of the above the number of standardized deaths is the sum of death rates by age-groups multiplied by standard weights. At the calculation of standardized death rates five-year*

age-groups of population were formed. The structure[1] of european standard population is as follows according to the WHO:

Age-group	*Population*
0	*1 600*
1– 4	*6 400*
5– 9	*7 000*
10–14	*7 000*
15–19	*7 000*
20–24	*7 000*
25–29	*7 000*
30–34	*7 000*
35–39	*7 000*
40–44	*7 000*
45–49	*7 000*
50–54	*7 000*
55–59	*6 000*
60–64	*5 000*
65–69	*4 000*
70–74	*3 000*
75–79	*2 000*
80–84	*1 000*
85–X	*1 000*
Total	*100 000*

Standardized death rates have been calculated with two decimal accuracy, as against the formerly employed one decimal, therefore there may be a slight discrepancy between the present and earlier publications.

***Standardized mortality ratio:** regional crude death rates calculated with the county age-structure and with the country-level five-year age group mortality rates of the given year expressed as a percentage of the country-level crude mortality rate. Five-year age-groups of counties population are taken as standard weights (if calculated for sexes, the population of the respective sex), while rates by age-groups are the ratio of deaths in the country and population of the relevant age-group.*

***Cause of death:** all those diseases, morbid conditions or injuries which either resulted in or contributed to death as well as the circumstance of an accident or violence which caused a fatal injury.*

***Underlying cause of death:** the cause of death selected for tabulation. The selection is carried out by applying the rules of the International Classification of Diseases.*

***Infant death:** death after live birth and before the completed age of one year. Still-born infant and those deceased on the anniversary of the birth are not counted among infant deaths.*

***Infant death rate:** infants deceased at the age under one year per 1000 live-born.*

***Net infant death rate:** it compares the dead infants grouped by age to the weighted sum of the live births occurred in the given quarter of two years.*

***Perinatal death:** the late foetal death and the infant death at the age of 0–6 days (early death of new-born) together.*

***Lost potential years:** the number of years not lived by the deceased from the potential lifetime of 0–70 years. For the age-groups over 70 years this rate is 0. In the yearbook the crude and standardized rates of the lost potential lifetime per hundred thousand concerned population (below the age of 70) are indicated. Standardization has been made according to the age-structure of the european standard population.*

---

[1] Source: WHO Europen health for all database (WHO Regional office for Europe, Copenhagen)

Average life expectancy: *expresses how many further years of lifetime can be expected by people of various age at the mortality rate of the given year.*

The calculation method of average life expectancy changed, since formerly the Becker-Zeuner method was used, while at present we use the Böck method and for equalization a biquadratic function is employed, replacing the former cubic function of less accuracy.

At the grouping of the regional data *till 1970 the basis of the grouping was the last permanent place of the residence of the deceased, from 1971 on his/her last actual place of residence. In case of the deceased who have only a place of residence (permanent registered dwelling) and have no place of stay (temporary registered dwelling) this place of residence is the administrative unit where they have or had, respectively, their last place of residence, in case of those who beside their place of residence (permanent registered dwelling) also have a place of stay (temporary registered dwelling) the administrative unit where they have or had, respectively, their last place of stay.*

The grouping by causes of death *was prepared on basis of the detailed list (A00–Y98) of the Xth revision of the International Classification of Diseases – ICD-X. The retrospective data were revised on basis of the ICD-X.*

In the former practice the classification of causes of deaths to the appropriate ICD category and the selection of the underlying cause were performed by a traditional (manual) method. During processing the 2005 cause of death data we turned to the automated data processing for the first time. The software we use is recommended by the European Union and it has been adapted to the national specialities. The methodological change slightly modified the structural composition of causes of death and at the same time in given causes of deaths it altered the earlier developed decennial trends. The methodological annex published on the CD provides detailed information about the reasons and the possible impacts.

The entry numbers of the following combination of the accidents (tables 1.6.9., 2.6.17–2.6.26., 2.6.29–2.6.30.) according to ICD-X are as follows: Railway accidents: V05, V15, V80.6, V81, V87.6, V88.6; motor vehicle accidents: V02–V04, 09.0, V09.2, V12–V14, V19.0-V19.2, V19.4-V19.6, V20-V29, V30-V39, V40-V49, V50-V59, V60-V69, V70-V79, V80.3-V80.5, V86, V87.0–V87.5, V87.7, V87.8, V88.0–V88.5, V88.7, V88.8., V89.0, V89.2; other vehicle accidents: V01, V06, V09.1, V09.3, V09.9, V10, V11, V16-V18, V19.3., V19.8, V19.9, V80.0-V80.2, V80.7–V80.9, V82-V85, V87.9, V88.9, V89.1, V89.3, V89.9, V98, V99; water vehicle accidents: V90–V94; air vehicle accidents: V95–V97; other and elsewhere non specified accidents: W35–W45, W50-W52, W88–W94, W99, X34–X39, X50–X59, Y40–Y84.

From 2005 the classification of the nature of injury *(tables 2.6.27. and 2.6.28.) is carried out by considering all injuries reported on the Death Certificate.*

Source of data: *complete accounting of deaths based on the data collections of the Hungarian Central Statistical Office performed on the Death Record, Certificate on the Examination of the Deceased. Notice Regarding Modification of the Death Certificate. Notice on Perinatal Death (pathological histology). Certificate on the Examination of the Perinatal Deceased according to §.10 of the 1993 Act No XLVI.*

INTERNAL MIGRATION AND RESIDENTIAL MOBILITY

(Tables 1.7.1–1.7.11., 2.7.1.–2.7.33., 3.1.11.–3.1.13., 3.7.1.–3.7.5.)

Internal migration follows the spatial, geographical movement of the population within the country. The basis of the observation is the system of dwelling registration concerning the permanent or temporary change in the place of residence. The statistical processing of migrations does not cover those forms of spatial mobility to which the obligation to register the dwelling does not refer: to commuting, staying in hotels-, holiday-homes-, hospitals-, sanatoriums: to military service, staying in temporary place of residence less than 15 days; to changes in dwelling of foreign citizens staying in Hungary and of persons in an official delegation of less than 14 days.

The regulations in 1954 regarding the obligation to register the home address (Order No. 1/1954./I.9.) MI on the identification card. Part two: On the obligation of registration of moving in and out – and in connection with them the method and contents of the observation – changed as follows:

The obligation to register persons under 16 years ceased from 1 August 1956 on. Till 1 January 1971 data contain only those persons under 16 years who move with the supporter (or another adult, respectively). (The number of those under 16 years moving with supporter and another adult, respectively, is scarcely lower than the total number of moving persons under 16 years.)

From 1 January 1971 the obligation of registration was extended to those of 14–15 years too, the children under 14 years moving alone were not included in the data collection till 31 December 1974 (their number is very low).

According to the order No. 24/1974. /VI.6./ of the Council of Ministers from 1 January 1975 on a separate registration form was introduced for persons under 14 years.

According to the order No. 44/1983. /XI.20./ of the Council of Ministers from 1 January 1984 on a different "Registration Record" was introduced regardless of the age and the character of internal migration.

The 1993 new "Registration Record of Home Address" does not inquire about the cause of moving, therefore from that date tables combined with the purpose of migration could not be prepared.

***Permanent migration** (in case of grouping by sex and age - migrant): the change in the place of residence when the migrant leaving his/her place of residence indicates a dwelling in another settlement as another place of residence. The place of out-migration is the former place of residence, while the place of in-migration is the new place of residence of the migrant.*

***Temporary migration** (in case of grouping by sex and age - migrant): the change in the place of dwelling crossing municipal boundaries when the migrant maintaining the place of residence changes a dwelling and indicates a new dwelling as a place of stay as well when he moves from one place of stay to another place of stay. The place of out-migration is the place of residence of the migrant or the former place of stay, the place of in-migration is the new place of stay.*

***Temporary remigration** (in case of grouping by sex and age - migrant): the change in the place of residence when the migrant leaving the place of stay returns to the place of residence. The place of out-migration is the place of stay, the place of in-migration is the place of residence of the migrant.*

***Permanent residential moving:** changing of dwelling within the border of a settlement, when the mover leaves his original place of residence and moves in a new address.*

***Temporary residential moving:** changing of dwelling within the border of a settlement, when the mover keeps its own dwelling and registers the new dwelling as place of stay, or if he moves from one place of stay to another, as well as when the mover, giving up his place of stay moves back to the place of residence.*

***Residential mobility:** changing of the dwelling within the administrative border of a settlement. In Budapest moving within or accross districts belong also here.*

***Total migration and total mobility rates:** they show if the recent conditions of migration and mobility prevailed, how many times a man in his lifetime would migrate or move*. Its calculation method: *the number of migrants and movers by ages is distributed by the corresponding mid-year population, and the sum of these ratios represent the number of per capita average migrations and moving*.

Migration and mobility rates have been calculated for mid-year population per thousand population.

Of the groupings: *The data on migrants by age are indicated on basis of the processing by year of birth, so they refer to years.*

Source of data: *the basis of the accounting of internal migration statistics is the registration system of home addresses on which the Central Data Processing, Registration and Electoral Office of the Ministry of the Interior supplies data sets for processing.*

INTERNATIONAL MIGRATION

(Tables 1.8.1.–1.8.19., 2.8.1.–2.8.38., 3.8.1.–3.8.22.)

The international migration statistics contain data on foreigners entering (migrating in), leaving (migrating out) the country, asylum-seekers and on those who were nationalized.

***Foreign citizen immigrating to Hungary**: the total number of foreign citizens asking for a residence permit or an immigration permit. In the registration the time of arriving in Hungary is originally unknown in numerous cases. From 1995, where it was unambiguous – in twenty per cent of the cases – we concluded what may be the likely time of entry by considering the first time of registration. There was not such an estimate before 1994.*

***Foreign citizen emigrating from Hungary**: a foreign citizen having a residence permit, an immigration permit or a settlement permit, who left Hungary without intending to return, or who have not prolonged their invalid permission and do not want to get a new one or people whose permit was invalidate by authority because of different causes.*

***Foreign citizen residing in Hungary**: a foreign citizen having a residence permit, an immigration permit, or a settlement permit who stayed in Hungary on 1 January of the given year. In the last year of the time series there is a fall of more than forty thousand, which has only administrative reasons. Since 1 January 2001 people with invalid residence permits do not belong to the foreign citizens residing in the country.*

***Refugee:** a foreign citizen or displaced person who for racial or religious reasons or because of his nationality or belonging to a certain group of society or as a result of his political views is pursued or has good reasons to fear pursuit in his home country, and consequently doesn't live in the country of his citizenship or in the case of a displaced person in the country of his usual residence, but is staying in the Hungarian Republic and cannot or doesn't wish to have the shelter of that country for fear of pursuit, provided that at his request the person concerned has been acknowledged refugee by the authority of refugee affairs.*

***Person granted subsidiary protection status:** mean a person who is provided temporary shelter and may not be returned to his home country, or in the case of stateless person to the country of domicile, for fear of being subjected to capital punishment, torture or any other form of cruel, inhuman or degrading treatment, and there is no safe third country offering refuge.*

***Person naturalised in Hungary**: someone who became a Hungarian citizen by naturalisation (he was born as a foreign citizen) or by denaturalisation (his former citizenship was abolished).*

***Hungarian citizen emigrating from Hungary**: a person who leaves Hungary for settling down permanently abroad and who announces it to the Ministry of the Interior.*

***Hungarian citizen returning:** a Hungarian citizen who was born abroad or has lived abroad and returned to Hungary in order to settle down.*

***Hungarian citizen born abroad:** persons born abroad as Hungarian citizens.*

***Residence permit**: from 1 January 2002 residence permits are no more classified as permanent or temporary permits. At the request of foreigners residing in Hungary with a valid residence visa - in order to extend the period of residence – the regional authority of aliens administration can issue residence permits.*

If the purpose of residence is working or some kind of activity in order to get an income, the residence permit can be issued for a maximum of four years for the first time. The validation period of the residence of foreigners studying in higher education or taking part in a further vocational training or in a professional practice can't exceed a year for the first time, and can be prolonged every time by a year until the studies or professional practices have been finished.

***Immigration permit:** until 31 December 2001 an immigration permit could be issued to a foreigner who resided in Hungary continuously and legally for at least three years since his entry, and had a permanent address and secure job in Hungary and there were no legal precluding reasons against him.*

***Permit to settle down**: (Settlement permit) it was introduced on January 1 2002 instead of the immigration permit. A foreigner can a residence permit if he has stayed in Hungary continuously legally and lived his life here for at least three years since his entry, except when the purpose of residence is continuing studies.*

A foreigner having a residence visa or residence permit can get an exemption from the condition of the three years' residence if he asks for residence as a family member with a purpose of family reunion.

Migration balance: *the difference between the number of immigrating and emigrating foreign citizens within a given year. If the number of immigrants is greater than the number of emigrants, the balance is positive, and if the number of emigrants is greater than the number of immigrants, the balance is negative.*

Calculation of the migration balance: *V_k=B-K (V_k = the calculated migration balance; B= the number of immigrating foreigners; K=the number of emigrating foreigners).*

In the tables we used the names of countries instead of the citizenship.

There is a difference in the tables containing the data of foreign citizens emigrating Hungary (1.8.9., 2.8.8., 2.8.9., 2.8.10.): the citizenship of those having entered from the Soviet Union, Czechoslovakia and Yugoslavia before 1993 was estimated by the place of their last foreign residence or the place of birth, respectively, progeny for citizenship of countries.

Till 1992 Yugoslavia means the Federal People's Republic of Yugoslavia, from 1993 the Federal Republic of Yugoslavia and from 2004 the Serbia and Montenegro.

The data processing method of the Ministry of Interior relating to the registration of foreigners changed by 1 January 2000. Retrospective data not comparable, since 1995 the processing was carried out by the different method. The Ministry of the Interior established a new register for EEA Nationals from 1. May 2004. The Hungarian Central Statistical Office have got a status of the register for the first time in November 2005. consequently the data for 2004 are incomplete regarding the EEA citizens.

Of the groupings: *the data on migrants by age have changed, from 1995 completed age is taken as basis.*

Source of data: *the registers of the foreigners from the Aliens Policing department and the annual reports of the Refugee affairs department of the Ministry of the Interior, Office of Immigration and Nationality, and the Register of personal data and addresses of the Central Data Processing, Registration and Election Office of the Ministry of the Interior.*

INTERNATIONAL DATA

(Tables 1.9.1.–1.9.15.)

Definitions used in this Chapter correspond to those in the specific Chapters containing Hungarian data.

Sources of international data have been the listed publications and data bases of international organizations and national statistical institutes:

Demographic Yearbook (UNO, New York)
Demographic Yearbook, Historical Supplement 1948-1997 (UNO, New York)
Recent demographic developments in Europe (Council of Europe, Strasbourg)
Statistics in focus (Eurostat, Luxembourg)
World Health Statistics Annual (WHO, Geneva)
NewCronos, Eurostat's Reference Database (Eurostat, Luxembourg)
WHO Statistical Information System, on-line database (WHO, Geneva)
United Nations Common Database (UNO, New York)
Monthly Bulletin of Statistics on-line (UNO, New York)
WHO European health for all database (WHO Regional office for Europe, Copenhagen)
World Mortality in 2000: Life Tables for 191 Countries (WHO, Geneva)
Web-sites of national statistical institutes
